# Development and Evaluation of Taste Masked Granular Formulation of Satranidazole by Melt Granulation Technique

**DOI:** 10.1155/2014/789676

**Published:** 2014-02-12

**Authors:** Harshal Ashok Pawar, Pooja Rasiklal Joshi

**Affiliations:** ^1^Department of Quality Assurance, Dr. L. H. Hiranandani College of Pharmacy, Smt. CHM Campus, Opp. Ulhasnagar Railway Station, Ulhasnagar, Maharashtra 421003, India; ^2^Dr. L. H. Hiranandani College of Pharmacy, Ulhasnagar, Maharashtra 421003, India

## Abstract

Drugs from nitroimidazole category are generally bitter in taste. Oral formulation with bitter taste is not palatable. Geriatrics and pediatrics patients usually suffer from swallowing difficulties. Many other patients in some disease conditions avoid swallowing tablets. Satranidazole is a new nitro-imidazole derivative with bitter taste and is available in market as film coated tablet. The purpose of this research was to mask the bitter taste of Satranidazole by coating complexation with low melting point wax and Eudragit EPO. Different types of wax (glyceryl monostearate, stearic acid and cetyl alcohol) were tried for taste masking. The drug to stearic acid ratio 1 : 2 was found to be optimum on the basis of taste evaluation and *in vitro* release. The formulated granules were found to possess good flow property. FTIR studies confirmed that there was no interaction between drug and excipients. Scanning Electron Microscopy of drug and the optimized batch of granules was performed. The *in vitro* release of drug from granules was compared with marketed tablet formulation. The taste masked granules of optimized batch showed 87.65% release of drug in 1 hr which is comparable to that of marketed tablet formulation.

## 1. Introduction

Masking the bitter taste of drugs is a challenge in development of all oral formulations and a necessity to ensure better patient compliance and product value where the process and formulation should be economic, rapid, and easy, involving least number of equipment, and processing steps and minimum number of excipients without adverse effect on drug bioavailability [1]. Bitter taste of drugs is a major problem in pediatric and geriatric formulations. Numerous methods are employed for effective taste masking, for example, use of flavors and sweeteners, microencapsulation, complexing with ion exchange resin, use of insoluble prodrug, formation of inclusion complexes, gelation, liposome, multiple emulsions, granulation, and so forth.

Taste masking can also be carried out using melt granulation technique [2]. Melt granulation is a process by which pharmaceutical powders are efficiently agglomerated by the use of a binder which can be a molten liquid or a solid that melts during the process. Different types of low melting point wax like glyceryl monostearate, palmitic acid, myristic acid, and so forth can also be used for taste masking as they form dense coating. Eudragit E100 is utilized along with low melting point waxes for dissolving it and spray cooled and granulated in a spray drying apparatus to obtain dense coating whereas if Eudragit E100 when dissolved in organic solvent forms porous coating which is difficult to mask the unpleasant taste [3].

Satranidazole (STZ) is a new nitroimidazole derivative with potent antiamoebic action. It is used in the treatment of intestinal and hepatic amoebiasis, giardiasis, trichomoniasis, and anaerobic infections. Its dose is 300 mg twice daily for 3–5 days in the treatment of amoebiasis and 600 mg as a single dose in the treatment of giardiasis and trichomoniasis. It is reported that Satranidazole exhibits significantly higher plasma concentrations than metronidazole and has a plasma elimination half-life of 1.01 hour which is significantly shorter than the corresponding metronidazole half-life of 3.62 hour [4]. Also, Satranidazole is having better tolerability, absence of neurological, and disulfiram-like reactions and it can be preferred in patients with susceptible neurological symptoms [5].

The present study aims for the development, optimization and evaluation of Satranidazole taste masked granular formulation using different types of low melting point wax with Eudragit EPO as a functional polymer by melt granulation technique.

## 2. Materials and Methods

### 2.1. Materials

Satranidazole was obtained as a gift sample from Alkem Laboratories, Mumbai. Eudragit EPO was obtained from Evonik Degussa, Mumbai. Satrogyl tablets (strength: 300 mg) were purchased from local market. All the chemicals and reagents used were of analytical grade.

### 2.2. Preformulation Studies

UV and FTIR spectrum of Satranidazole were taken to confirm the identity of drug. Compatibility study between the drug and the excipients was done using FTIR. IR spectra were recorded in Fourier Transform Infrared spectrophotometer (Shimadzu Corporation) with KBr pellets.

### 2.3. Development and Optimization of Granular Formulation

In preliminary study, three different trial batches of granules were prepared using glyceryl monostearate, stearic acid, and cetyl alcohol. The purpose of these trials was to select the wax suitable for taste masking of Satranidazole in low concentration since the dose of the drug is high. The required quantity of wax was weighed and melted in porcelain dish. Eudragit EPO was dissolved in molten wax and drug was added followed by mixing. At slightly lower temperature all the other ingredients were added and mixed. Finally the mixture was allowed to cool slightly and the solidified mass was passed through 12 # sieve and then through 16 #. The granules obtained were then lubricated with flavorant, sweetener and lubricant. The composition of granules is shown in Table 1.

Different batches of granules were prepared using wax selected in above trials to optimize drug to ratio for successful taste masking of STZ. The composition of the formulated batches is as shown in Table 2.

### 2.4. Evaluation of Granules

The prepared batches were evaluated for the following parameters.

#### 2.4.1. Flow Properties

The granules were evaluated for following flow properties using following parameters [6, 7].

#### 2.4.2. Angle of Repose

Fixed funnel method was used to determine angle of repose and was calculated using following equation:

(1) $$\begin{array}{c} \tan\theta =\frac{h}{r}, \end{array}$$

where *h* is height and *r* is radius.

#### 2.4.3. Bulk Density and Tapped Density

To measure density, the granules were filled in a 100 mL capacity measuring cylinder up to at least 3/4th the height. Bulk density is the quotient of weight to the volume of the sample. Tapped density is the quotient of weight of the sample to the volume after taping a measuring cylinder 500 times from a height of ~1.5 in.

#### 2.4.4. Hausner Ratio and Carr's Index

Hausner's ratio was calculated using the following formula:

(2) $$\begin{array}{c} \text{Hausner's Ratio}=\frac{\text{Tapped}\;\text{Density}}{\text{Bulk}\;\text{Density}}. \end{array}$$

The percentage compressibility (Carr's index) was calculated as 100 times the ratio of the difference between tapped density and bulk density to the tapped density:

(3) $$\begin{array}{c} \text{Carr's index}=\frac{\left(\text{Tapped}\;\text{Density}-\text{Bulk}\;\text{Density}\right)}{\text{Tapped}\;\text{Density}}\times 100. \end{array}$$

#### 2.4.5. Granular Friability

Ten grams of uncoated granules was subjected to friabilator at 25 rpm. After 4 minutes, the granules were sieved on a 200 mesh. The amount of granules passed through 200 mesh was calculated as percentage granular friability.

#### 2.4.6. Particle Size Distribution

Particle size distribution was performed on optimized batch using nest of standard sieves (20 #, 40 #, 60 #, 100 #, and 120 #). The sieves were agitated mechanically for 10 minutes on a sieve shaker and the weight of granules retained on each smaller sieve was noted.

#### 2.4.7. Scanning Electron Microscopy (SEM)

The surface morphology of uncoated and coated granules was examined using a scanning electron microscope (Zeiss Ultra Plus-FESEM). The samples of granules were previously sputter-coated with gold.

#### 2.4.8. *In Vitro* Dissolution Studies in 0.1 N Hydrochloric Acid (HCl)

*In vitro* dissolution studies were carried out using USP II Apparatus (Paddle Method) rotating at 75 rpm in 900 mL of 0.1 N HCl as dissolution media maintained at 37 ± 0.5°C. Sampling was done at different time intervals of 10, 15, 30, 45, and 60 minutes by withdrawing 5 mL of the dissolution medium and replacing it with the same amount of medium to maintain sink conditions. The withdrawn samples were filtered and the contents of sample were determined spectrophotometrically at 320 nm. The dissolution of marketed tablet formulation (Satrogyl Tablet, 300 mg) was also carried using the similar dissolution conditions. The percent cumulative release of drug was calculated using standard calibration curve of STZ prepared in 0.1 N HCl.

#### 2.4.9. Determination of Drug Content (Percent Assay) Using HPLC

The assay of different batches of granules of Satranidazole with stearic acid was carried out using previously developed and validated HPLC method. Isocratic elution at a flow rate of 1.0 mL/min was employed on BDS Hypersil C18 (250 mm × 4.6 mm, 5 *μ*m) column at 25°C temperature. The mobile phase consisting of 0.16% v/v orthophosphoric acid solution, pH 3, and acetonitrile in the ratio of 60 : 40 v/v was used. The UV detection wavelength was 320 nm, and 20 *μ*L sample was injected. Standard stock solution was prepared by dissolving 50 mg of Satranidazole in 50 mL methanol and was further diluted with mobile phase to obtain standard solution of 40 *μ*g/mL concentration. The sample solution was prepared by adding granules equivalent to 20 mg of Satranidazole in methanol. The dispersion was sonicated for 30 min and was then filtered. The resultant solution was further diluted with mobile phase to get 40 *μ*g/mL of test solution. The solutions were filtered through 0.45 *μ* nylon filter. Equal volumes of sample preparation and standard preparation were injected separately into the HPLC (an agilent high performance liquid chromatograph equipped with quaternary pump) and chromatograms were recorded.

### 2.5. Taste Masking Evaluation

#### 2.5.1. Determination of Bitter Taste Recognition Threshold of Satranidazole

Threshold value of STZ was determined based on the bitter taste recognized by eight volunteers in the age group of 21–28 years. Aqueous solutions of STZ with different concentrations (10, 20, 30, and 40 *μ*g/mL) were prepared. One milliliter of solution was placed on the center of the tongue of volunteer for 30 seconds. The solution was spat out after 30 seconds, and the mouth was thoroughly rinsed with distilled water. The same procedure was repeated for all solutions and volunteers. A gap of 30 minutes was maintained in between tasting two different solutions. The same procedure was repeated for STZ solutions with concentrations 24, 26, 28, 32, and 35 *μ*g/mL. The threshold value was selected on the basis of the lowest concentration that had a bitter taste [8–10].

#### 2.5.2. *In Vitro* Evaluation of Bitter Taste of Granules

Granules equivalent to 25 mg of STZ were placed in a volumetric flask with 50 mL of phosphate buffer (pH 6.8) and stirred for 5 minutes. The mixture was filtered, and the filtrate was analyzed for STZ concentration at 320 nm by UV-Visible spectrophotometer and that was compared with the threshold value [11].

#### 2.5.3. *In Vivo* Taste Evaluation

Two procedures were used for *in vivo* taste evaluation.

1. Gustatory sensation taste: informed consent was first obtained from 8 healthy volunteers and taste evaluation study was carried out. The whole dose was added to 100 mL of water for 15 seconds. STZ was used as control. After 15 seconds, 1 mL of dispersion was held in the mouth of each volunteer for 30 seconds and then spat out [8]. The bitterness level was recorded by using the numerical scale shown in Table 3.
2. Granules equivalent to 50 mg STZ were held in mouth of each volunteer for 30 seconds. After expectoration, bitterness level was recorded by using the numerical scale shown in Table 3.

### 2.6. Stability Studies

The optimized granular formulation was subjected for stability study for one month according to ICH guidelines. Tests were conducted at room temperature (RT) and accelerated stability conditions. The samples were designated as time 0 and 1 month for RT and 0 and 1 month for accelerated studies. Samples designed for RT storage were kept at 25 ± 2°C and 60 ± 5% relative humidity (RH). The samples in the accelerated stability study were kept at 40 ± 2°C and 75 ± 5% RH in humidity chamber. Samples were tested for its appearance, flow properties, taste masking, *in vitro* release, and drug content using the previously described procedures.

## 3. Results and Discussion

**Figure 1:**
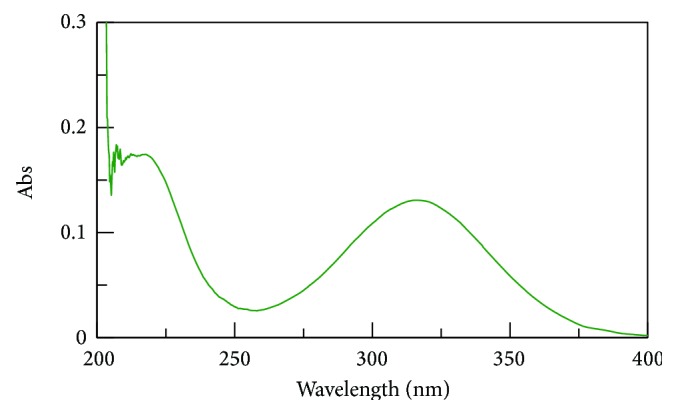
UV spectrum of STZ in methanol.

**Figure 2:**
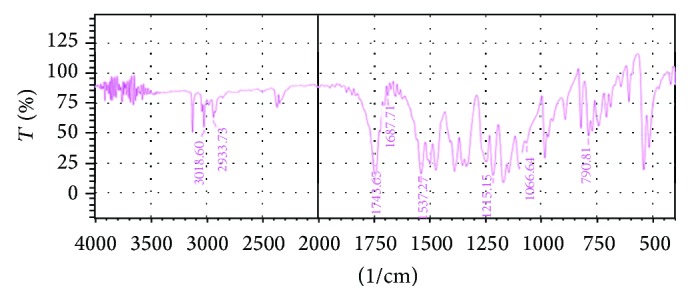
FTIR of STZ.

**Figure 3:**
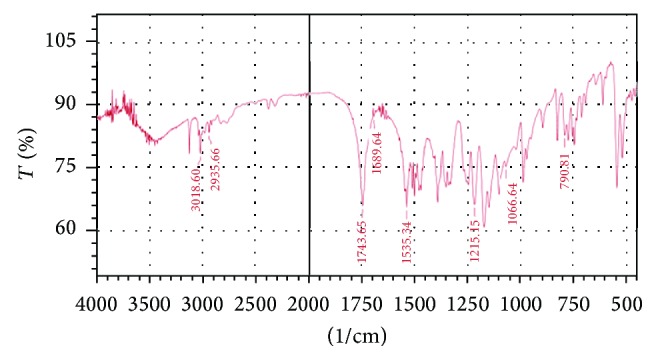
FTIR of drug with Eudragit EPO.

**Figure 4:**
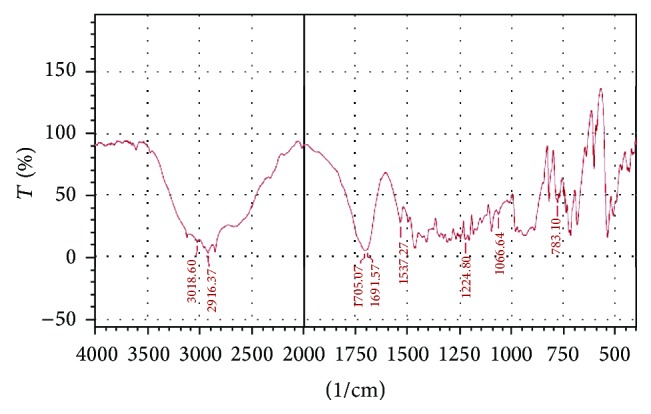
FTIR of STZ with Stearic acid.

**Figure 5:**
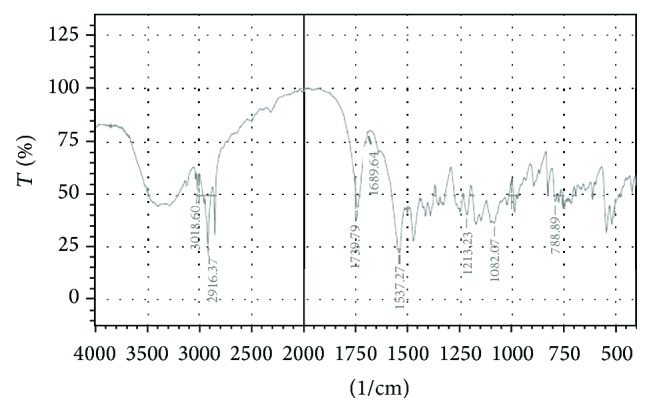
FTIR of optimized formulation (F3).

The UV spectrum of STZ in methanol is shown in Figure 1. The *λ* max of Satranidazole sample was found to be 320 nm. STZ exhibited characteristic peaks at 1687, 1743, 1066, 1537, and 1215 cm^−1^ attributed to C=N stretching, C=O stretching, S=O stretching, C–NO_2_ stretching, and C–N vibrations. FTIR spectra showed all the important peaks of drug in spectra of drug-excipient mixture and formulation. FTIR of STZ, physical mixture of STZ with Eudragit EPO and stearic acid, and the optimized formulation is represented in Figures 2, 3, 4, and 5, respectively.

All the excipients were selected based on preformulation study results and extensive literature survey. Low melting point wax was used to form dense coating around the drug particle to mask the taste. Eudragit EPO was used as a functional polymer which is insoluble above pH 5 and thus prevents release of STZ in mouth. Preliminary trials were taken for the selection of wax. The ratio of the drug to wax was selected based on literature survey [2, 3]. Initially, three different granular formulations were prepared using three different types of wax (glyceryl monostearate, stearic acid, and cetyl alcohol) individually in drug to wax ratio 1 : 2. In preliminary trials, it was found that granules of STZ formed with stearic acid (Trial 2) were better taste masked. The granules formed with glyceryl monostearate and cetyl alcohol in the same ratio with drug were bitter in taste. Hence, the ratio of STZ to stearic acid was varied from 1 : 1 to 1 : 2.5 in further optimization and formed batches were evaluated for taste masking and other parameters such as flow property, drug content (assay), and *in vitro *drug release.

The results of bulk density, tapped density, Carr's index, Hausner ratio, and angle of repose are summarized in Table 4. The results indicated that the prepared granules possess good flow property [12].

The taste recognition threshold of STZ was determined on the basis of Table 5. The threshold was found to be 28 *μ*g/mL.

Under *in vitro* taste masking evaluation study, the drug release in pH 6.8 phosphate buffer was studied. It was observed that the granules formed with glyceryl monostearate were showing more release than threshold concentration in 6.8 phosphate buffer and were less hard and the granules formed with cetyl alcohol were found to be very hard as cetyl alcohol used to quickly solidifies on melting and therefore the drug was not completely coated with it and showed more release than threshold concentration in 6.8 phosphate buffer.

Results of drug release from granules of batches F1 and F2 were found to be more than the threshold value, that is, 28 *μ*g/mL. The drug release from granules of batches F3 and F4 was found to be less than the threshold value which indicated successful taste masking of granules. Gustatory sensation test results obtained from 8 healthy volunteers also indicated that the granules of batches F3 and F4 were better taste masked.  Table 6 indicates the *in vivo* and *in vitro* taste masking evaluation results of the granular formulations F1 to F4.

**Figure 6:**
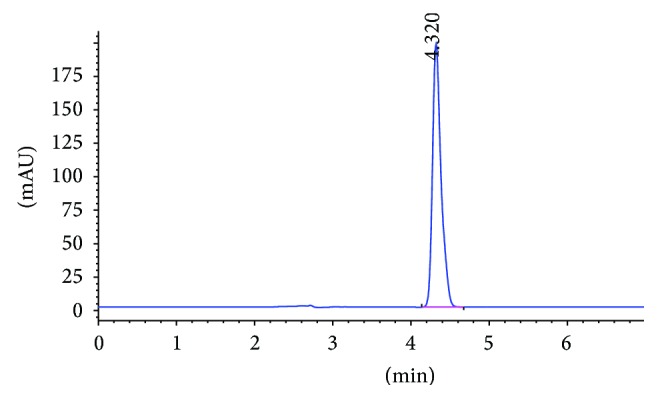
Typical chromatogram of test solution.

Typical HPLC chromatogram of test solution is represented in Figure 6. The STZ peak was eluted at the retention time of about 4.32 minutes. The percentage of drug content for different batches (F1–F4) was found to be in the range of 98–102%.

**Figure 7:**
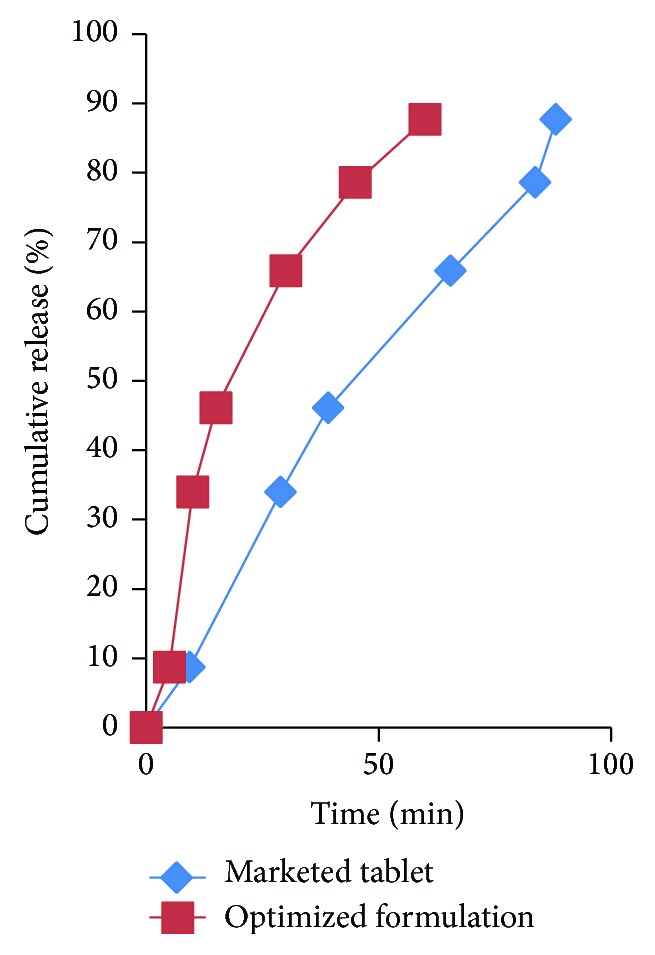
Comparison of *in vitro* dissolution study of optimized formulation and marketed tablet in 0.1 N HCl.

The results of *in vitro* dissolution studies and drug content (assay) of different batches (F1–F4) are summarized in Table 7. It was found that as the amount of stearic acid increases in the granules and the % drug release in 0.1 N HCl decreases, the granular strength increases and the friability decreases. The batch F4 formulated with drug to stearic acid ratio 1 : 2.5 showed 76.87% release in 0.1 N HCl, whereas batch F3 showed almost 87.65% release of drug in 1 hr which was almost same as that of the marketed tablet, that is, 88.1% release in 1 hr. The formulated granules with drug to stearic acid ratio 1 : 2 showed better release in 0.1 N HCl with complete taste masking. Hence, formulation F3 was considered as optimized formulation. Batch F3 was subjected to stability studies for one month as per ICH guidelines. The comparison of *in vitro* drug release from optimized formulation (F3) and marketed tablet in 0.1 N HCl is shown in Figure 7.

**Figure 8:**
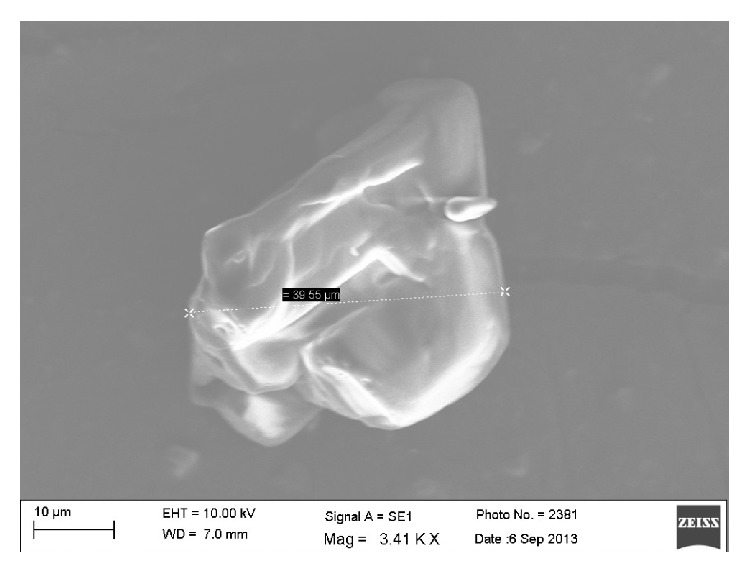
SEM image of drug particle.

**Figure 9:**
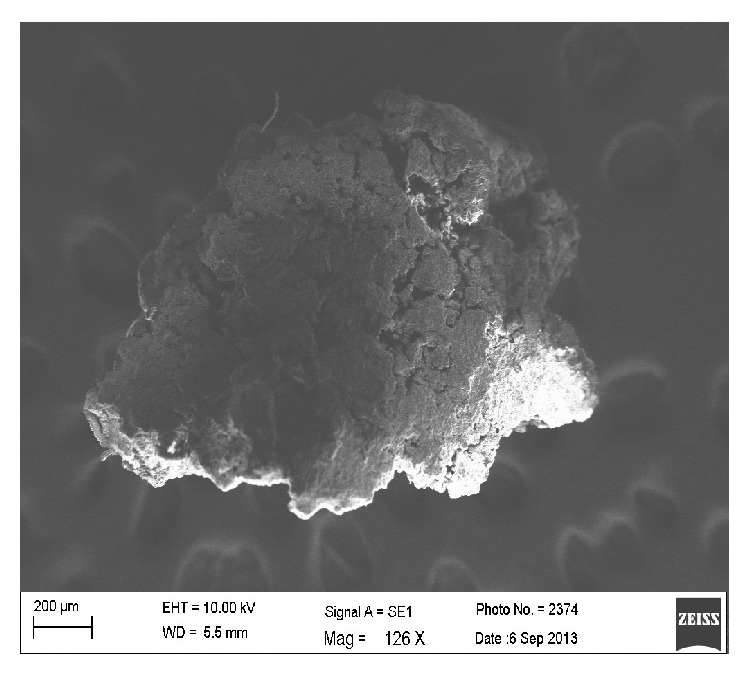
SEM image of optimized granular formulation (F3).

The Granular friability of granules of optimized batch (F3) was found to be 0.15%. The size of the granules was found in the range of 341.93 *μ*m–487.5 *μ*m. SEM photographs of drug and granules are as shown in Figures 8 and 9, respectively. It was found that the surface of the drug particle was smooth and that of the granules was rough.

Stability study results indicated that there was no change in physical appearance of granules and taste at room temperature as well as accelerated conditions. The results of percent dissolution, assay, and flow properties of the optimized batch F3 at RT and accelerated stability conditions are summarized in Table 8. The optimized formulation was found stable during stability study.

## 4. Conclusion

Taste masking of bitter drugs with higher dose is challenging. STZ is having a dose of 300 mg twice daily in amoebiasis and is available in market in the form of film coated tablet. Difficulty in swallowing tablets is a major problem especially in case of geriatrics and pediatrics as well as patients who are not able to swallow tablets. In the above technique, low melting point wax was used for dissolving Eudragit EPO instead of organic solvent which led to the formation of dense coating around the drug and also step of removal of organic solvent by carrying out drying step was not required by using wax. Granules formed by coating with Eudragit EPO showed similar release of STZ like that of marketed tablet. From the above results, it can be concluded that complete taste masking of bitter STZ is achieved by granulating drug with stearic acid and Eudragit E100 by melt granulation technique. The efficiency of stearic acid was tested for the purpose of taste masking with the possible method of melt granulation. Taste masking by the above technique is achieved by decreasing the surface area of the drug by increasing its particle size and the method was found to be very simple.

## Figures and Tables

**Table 1:** Composition of preliminary trial batches.

Ingredients	Batches			Role
	Trial 1	Trial 2	Trial 3	
	Quantity in mg per dose		
Satranidazole	300	300	300	Active ingredient
Glyceryl monostearate	600	—	—	Low melting point wax
Cetyl alcohol	—	—	600	Low melting point wax
Stearic acid	—	600	—	Low melting point wax
Eudragit EPO	100	100	100	Functional polymer
Starch 1500	200	200	200	Disintegrant
Magnesium oxide	70	70	70	Additive for taste masking
Mannitol	498	498	498	Diluent
Xylitol	42	42	42	Sweetener
Sodium carboxymethyl cellulose (sodium CMC)	20	20	20	Disintegrant
Hydroxyllpropyl cellulose (low substituted) HPC	80	80	80	Disintegrant
Vanilla	20	20	20	Flavorant
Aspartame	65	65	65	Sweetener
Magnesium stearate	5	5	5	Lubricant
*In vitro *taste evaluation by UV (*μ*g/mL)	37.81	23.33	33.14	—

^*^All the batches showed less than threshold value in *in  vitro* taste evaluation by UV.

**Table 2:** Composition of batches prepared using selected wax for optimization.

Ingredients	F1	F2	F3	F4
	Ratio of drug : stearic acid			
	1 : 1	1 : 1.5	1 : 2	1 : 2.5
	Quantity in mg per dose			
Satranidazole	300	300	300	300
Stearic acid	300	450	600	750
Eudragit EPO	100	100	100	100
Starch 1500	200	200	200	200
Magnesium oxide	70	70	70	70
Mannitol	798	648	498	348
Xylitol	42	42	42	42
Sodium CMC	20	20	20	20
HPC	80	80	80	80
Vanilla	20	20	20	20
Aspartame	65	65	65	65
Magnesium stearate	5	5	5	5

**Table 3:** Numerical scale for bitterness level.

Score	Inference
0	Pleasant
1	Tasteless
2	Slightly bitter
3	Moderately bitter
4	Extremely bitter

**Table 4:** Results of flow evaluation.

Parameters	F1	F2	F3	F4
Bulk density (gm/cm^3^)	0.50 ± 0.028	0.50 ± 0.031	0.52 ± 0.031	0.53 ± 0.021
Tapped density (gm/cm^3^)	0.57 ± 0.026	0.56 ± 0.015	0.58 ± 0.013	0.60 ± 0.023
Carr's compressibility index (%)	12.281 ± 0.075	12 ± 0.102	10.345 ± 0.081	11.667 ± 0.098
Angle of repose (°)	29.34 ± 0.176	25.61 ± 0.081	25.42 ± 0.142	27.33 ± 0.133
Hausner ratio	1.14 ± 0.011	1.12 ± 0.009	1.1153 ± 0.01	1.1320 ± 0.01

^*^All above readings are average ± standard deviation, *n* = 6.

**Table 5:** Taste recognition threshold determination.

Concentration (*μ*g/mL)	Volunteers							
	1	2	3	4	5	6	7	8
10	N	N	N	N	N	N	N	N
20	N	N	N	N	N	N	N	N
22	N	N	N	N	N	N	N	N
24	N	N	N	N	N	N	N	N
26	N	N	N	N	N	N	N	N
28	N	N	N	Y	N	Y	N	N
30	Y	N	Y	N	Y	Y	Y	Y
32	Y	Y	Y	Y	Y	Y	Y	Y
35	Y	Y	Y	Y	Y	Y	Y	Y
40	Y	Y	Y	Y	Y	Y	Y	Y

^*^Y: recognition of bitter taste; N: no perception of bitter taste.

**Table 6:** Results of *in vivo* and *in vitro* taste masking evaluation.

Batch	Taste masking score by gustatory sensation test	*In vitro* release of STZ by UV in 5 min (*μ*g/mL)
F1	3	32.01
F2	2	30.97
F3	1	23.33
F4	1	21.12

**Table 7:** % cumulative release and assay in 0.1 N HCL.

Time (minutes)	F1	F2	F3	F4	Marketed formulation
5	7.61 ± 0.367	8.02 ± 0.512	8.71 ± 0.426	5.31 ± 0.378	9.34 ± 0.432
10	86.93 ± 0.523	52.99 ± 0.69	33.9 ± 0.299	25.71 ± 0.362	28.87 ± 0.471
15	98.46 ± 0.647	62.68 ± 0.489	46.05 ± 0.343	36.12 ± 0.298	39.1 ± 0.572
30	104.34 ± 0.701	73.40 ± 0.397	65.83 ± 0.378	58.54 ± 0.452	65.43 ± 0.503
45	102.17 ± 0.489	81.66 ± 0.434	78.57 ± 0.418	71.27 ± 0.514	83.66 ± 0.66
60	102.11 ± 0.585	90.01 ± 0.546	87.65 ± 0.404	76.87 ± 0.539	88.1 ± 0.55
Assay (%)	100.87 ± 0.465	98.91 ± 0.487	99.69 ± 0.574	101.25 ± 0.521	97.89 ± 0.464

^*^All above readings are average ± standard deviation, *n* = 6.

**Table 8:** Stability study results.

Parameters	At RT		At accelerated conditions	
	0 month	1 month	0 month	1 month
Assay (%)	98.94	98.75	98.87	98.62
% dissolution (in 1 hr)	85.01	84.70	84.68	84.97
Flow property	Good	Good	Good	Good

## References

[1] Khames A., Helmy A., Kady S., Abd-elbary A. (2011). Preparation, characterization, and *in-vitro/vivo* evaluation of indion-based chewable tablets of paracetamol and ibuprofen for pediatric use. *Journal of American Science*.

[2] Ahire S. B., Gaikwad P. D., Bankar V. H., Pawar S. P. (2012). Taste masking of metoclopramide hydrochloride by novel melt granulation. *International Journal of Drug Delivery*.

[3] Yajima T., Ishii K., Umeki N. Taste masking pharmaceutical composition.

[4] Pargal A., Rao C., Bhopale K. K., Pradhan K. S., Masani K. B., Kaul C. L. (1993). Comparative pharmacokinetics and amoebicidal activity of metronidazole and satranidazole in the golden hamster, Mesocricetus auratus. *Journal of Antimicrobial Chemotherapy*.

[5] Parmar D. M., Jadav S. P. (2007). The concept of personal drugs in the undergraduate pharmacology practical curriculum. *Indian Journal of Pharmacology*.

[6] Pawar H. A., D’mello P. M. (2011). Development and evaluation of herbal laxative granules. *Journal of Chemical and Pharmaceutical Research*.

[7] Remya K. S., Beena P., Bijesh P. V., Sheeba A. (2010). Formulation development, evaluation and comparative study of effects of super disintegrants in cefixime oral disintegrating tablets. *Journal of Young Pharmacists*.

[8] Albertini B., Cavallari C., Passerini N. (2004). Characterization and taste-masking evaluation of acetaminophen granules: comparison between different preparation methods in a high-shear mixer. *European Journal of Pharmaceutical Sciences*.

[9] Chang W., Chung J. W., Kim Y., Chung S., Kho H. (2006). The relationship between phenylthiocarbamide (PTC) and 6-n-propylthiouracil (PROP) taster status and taste thresholds for sucrose and quinine. *Archives of Oral Biology*.

[10] Gao Y., Cui F., Guan Y., Yang L., Wang Y., Zhang L. (2006). Preparation of roxithromycin-polymeric microspheres by the emulsion solvent diffusion method for taste masking. *International Journal of Pharmaceutics*.

[11] Bora D., Borude P., Bhise K. (2008). Taste masking by spray-drying technique. *AAPS PharmSciTech*.

[12] Shaik M., Arunachalam A., Sudhakar A. M. S., Vanitha N., Koteswararao C. H., Bhavani P. G. (2012). Invention and *in vitro* evaluation of floating tablets of metformin hydrochloride using hydrophilic polymer as release retardant. *International Journal of Biological and Pharmaceutical Research*.

